# Prenatal alcohol history – setting a threshold for diagnosis requires a level of detail and accuracy that does not exist

**DOI:** 10.1186/s12887-019-1759-1

**Published:** 2019-10-23

**Authors:** Susan Petryk, Muhammad A. Siddiqui, Juliet Ekeh, Mamata Pandey

**Affiliations:** 1 Developmental Paediatrician, Clinical Associate Professor, University of Saskatchewan, Child and Youth Services, 1680 Albert Street, Regina, Saskatchewan S4P-5A6 Canada; 2 Department of Research, Saskatchewan Health Authority, Regina, Saskatchewan Canada; 3 Department of Medicine, University of Nigeria Teaching Hospital, Ituku Ozalla, Enugu, Nigeria

**Keywords:** Prenatal alcohol exposure, Fetal alcohol Spectrum disorder

## Abstract

**Background:**

The revised 2015 Canadian Guidelines requires a more specific prenatal alcohol exposure (PAE) threshold for a Fetal Alcohol Spectrum Disorder (FASD) diagnosis. The unintended consequences of adhering to the suggested PAE threshold for an FASD diagnosis and the challenges professionals face in obtaining an accurate PAE history were explored.

**Methods:**

Using a mixed methods study design, the study was carried out in two parts (Quantitative and Qualitative). PAE history and FASD diagnosis was reviewed retrospectively from 146 patient charts referred for an FASD assessment between 2011 and 2016. The challenges experienced when collecting the PAE history were explored through interviews with 23 professionals. Statistical analysis was performed using SPSS (IBM SPSS Statistics 20.0).

**Results:**

Of 146 assessments, only 21.9% met the revised 2015 PAE guidelines while 79.4% met the previous 2005 PAE criteria. Of 146 clients, 54.1% met brain criteria for FASD yet of those only 29.1% met the revised PAE criteria whereas 70.9% did not and therefore could lose their FASD diagnosis under a diligent application of PAE level suggested in the 2015 Guidelines. Thematic analysis of the interview data indicated that obtaining a reliable PAE history was challenging and a combination of methods are employed to get credible information.

**Conclusion:**

Confirming PAE history can be difficult, but ensuring reliable and accurate details on quantity, frequency, and timing of exposure is impossible in a clinical setting. Three out of every four individuals in the present study lost their FASD diagnosis following implementation of 2015 Canadian FASD Guidelines. A threshold might also imply that alcohol consumption below threshold is safe. The 2015 Canadian Guidelines need further refinement regarding the PAE criteria.

## Background

A Fetal Alcohol Spectrum Disorder (FASD) diagnosis is made in individuals exposed to alcohol prenatally who also have a variety of severe and chronic developmental and behavioural impairments [1] In a recent meta-analysis, global prevalence of FASD was estimated to be 7.7 per 1000 population (95% CI, 4.9–11.7 per 1000 population), with the European Region having the highest overall prevalence at 19.8 per 1000 population (95% CI, 14.1–28.0 per 1000 population) and the Eastern Mediterranean Region having the lowest overall prevalence at 0.1 per 1000 population (95% CI, 0.1–0.5 per 1000 population) [2]. The prevalence estimates for FASD vary widely depending on setting, group studied and methodology of ascertainment [3] however FASD is known to be a leading cause of disability worldwide [1, 2, 4–6].

A diagnosis of FAS can be made without a PAE history only in the case where the 3 sentinel facial features are all present. Despite extensive research there still is no unique neurodevelopmental profile that distinctly differentiates FASD from other neurodevelopmental disorders. Therefore, a comprehensive physical and neurodevelopmental assessment made by a multidisciplinary clinical team is important to reduce false positives and conclusively diagnose FASD [2, 4, 7]. In Canada, the Canadian Guidelines for FASD Diagnosis and the University of Washington FASD 4-Digit Diagnostic Code are commonly used [8–10]. These two diagnostic systems have been harmonized and are often referred to interchangeably in Canadian settings.

A credible history of prenatal alcohol exposure (PAE) is required in almost all cases [8, 11]. According to the revised 2015 Canadian Guidelines a diagnosis may be made if there is “*confirmation of PAE with an estimated dose at a level known to be associated with neurodevelopmental effects”.* An appendix to revised 2015 Canadian Guidelines specifies that the *“threshold known to be associated with neurodevelopmental effects is 7 or more standard drinks per week, or any episode of drinking 4 or more drinks on the same occasion. Because the effect sizes seen with a single binge episode are relatively small, a threshold of 2 binge episodes is recommended as a minimum for diagnosis”.* The appendix acknowledges this threshold is tentative pending updated information.

By comparison, the FASD 4-Digit Diagnostic Code has two accepted levels of PAE confirmation; (a) PAE is consistent with the medical literature placing the fetus at “high risk” OR (b) PAE is confirmed but in lower amounts than above or exact amounts unknown. Clinicians are aware that PAE histories received in a clinical setting are often unreliable and detailed exposure history is unverifiable [7, 12]. Additionally, a proposed threshold creates several ethical and diagnostic problems such as precluding individuals from a diagnostic assessment, implicitly suggesting below threshold to be safe, and above the threshold as harmful always leading to a similar degree of harm for all, which a recent large twin study has disproved [13]. The purpose of the present study is to examine one of the unintended consequences of adhering to a specified minimum PAE threshold.

## Methods

Employing a mixed methods design the study was carried out in two parts.

### Part-1 quantitative

The objective of Part-1 was to examine the unintended consequences of adhering to a specific minimum PAE threshold for FASD diagnosis by retrospectively applying the 2015 Canadian guidelines for PAE to those already diagnosed with FASD recently.

Client charts (*N* = 146) referred to Child and Youth Services (CYS), Regina, Saskatchewan between 1^st^January-2011 and 30^th^November-2016 for FASD assessment were reviewed retrospectively to abstract basic demographics, PAE history and evaluations of brain criteria for an FASD diagnosis. This clinic reports both diagnostic labels from the Canadian Guidelines and the 4 Digit Diagnostic Codes.

The PAE history was divided into four categories consistent with the FASD 4-Digit Code: PAE Rank 4 is consistent with the medical literature placing the fetus at “high risk”, PAE Rank 3 is confirmed but in lower amounts than above or exact amounts unknown, PAE Rank 2 is unknown and PAE Rank 1 is confirmed absence of PAE. Within the new guidelines, only clients with a PAE Rank 4 would have a sufficient PAE for an FASD diagnosis and those with a PAE Rank 3 or lower would be below threshold so could lose their diagnosis.

The magnitude of structural and/or functional brain abnormalities were ranked following the FASD 4-Digit Code (see Fig. 3). The Canadian Guidelines and FASD 4-Digit Code were harmonized during this study period so brain criteria should essentially remain the same [12]. With the FASD 4-Digit Code, brain function rankings are classified by assessing up to nine developmental domains such as cognitive ability, achievement, memory, executive function, motor skills, language, attention and neurological “soft signs”. Statistical analysis was performed using SPSS (IBM SPSS Statistics 20.0). Descriptive statistics mean (SD) and percentages were calculated to answer the research questions.

## Results

Out of the 146 clients 97 (66.4%) were males, 49 (33.6%) clients were residing with foster parents, Attention deficit hyperactivity disorder (ADHD) was the top most common comorbidity diagnosed in 94 (65.28%) clients and 99 (67.8%) clients received pharmacological treatment (See Table 1).

**Table 1:** Demographic information of the clients (n-146)

Variables	Number (%)
Gender	
Male	97 (66.4%)
Female	49 (33.6%)
Mean age Female (*SD; SE*)*	10 (2.7;.4)
Mean age Male (*SD;SE*)	9.1 (2.6;.3)
Parental status	
Adopted	25 (17.1%)
Living with biological parents	30 (20.6%)
Living with foster parents	49 (33.6%)
Living with grandparents/ others extended family members/ family members or person with significant interest	34 (23.3%)
Living in foster homes / group homes	8 (5.48%)

*SD Standard Deviation*, SE* Standard Error

### Prenatal alcohol exposure history

**Fig. 1:**
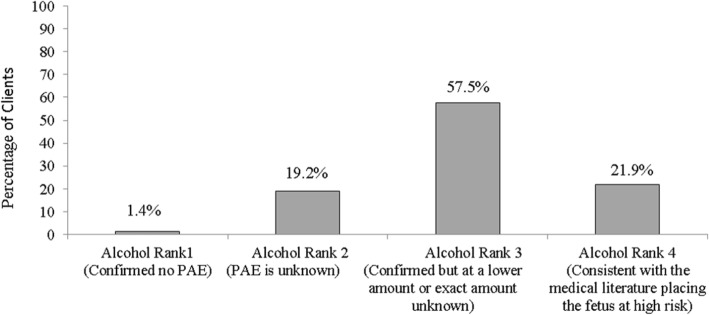
Percentage of referrals in which prenatal alcohol quantity and frequency is recorded in accordance with the FASD 4-Digit Code (*N* = 146)

Figure 1 indicates that of 146 clients, in the majority of clients, i.e. 84 (57.5%), PAE was confirmed but exact amount was unknown. Out of 146 clients 30 clients lived with biological parents and out of these 30 clients in 11 (36.7%) clients PAE was confirmed but the exact amount was unknown and in 13 (43.3%) PAE was reported to be consistent with the medical literature placing the fetus at high risk. A large number of clients in our sample were not residing with biological parents and therefore obtaining reliable PAE history was more challenging.

**Fig. 2:**
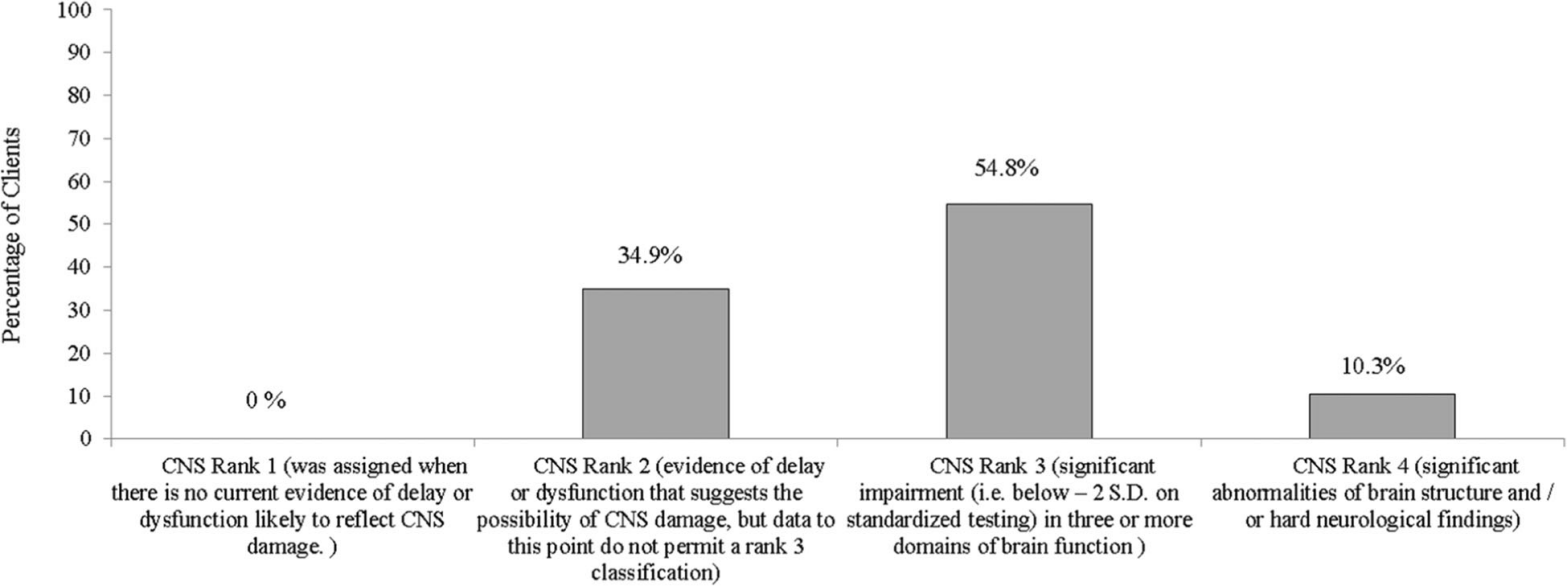
Severity of Brain damage/dysfunction in accordance with the FASD 4-Digit Code (*N* = 146)

Figure 2 indicates that of 146 clients, a substantially larger number or 80 (54.8%) clients showed significant impairment (i.e. below – 2 S.D. on standardized testing) in three or more domains of brain function.

### Combining the CNS and PAE rankings for an FASD diagnosis

Table 2 illustrates PAE and corresponding central nervous system (CNS) rank for clients. According to the 2005 FASD Guidelines a Brain Rank of 3 or 4 with a PAE Rank 3 or 4 is required for FASD diagnosis (with the exception that full FAS can be diagnosed with PAE Rank 2, i.e. unknown). Tables 2 and 3 show that the majority of clients with an FASD diagnosis in this study had a PAE Rank 3. Changing the PAE definition would therefore greatly diminish the number who gets an FASD diagnosis.

**Table 2:** CNS rank of clients who’s PAE score was 3 or 4

	Alcohol exposure =3 (*N* = 84)	Alcohol exposure =4 (*N* = 32)
CNS Rank 2	28 (33%)	9 (28.1%)
CNS Rank 3	50 (60%)	19 (59.4%)
CNS Rank 4	6 (7%)	4 (12.5%)

Eligible for FASD diagnosis Canadian guidelines 2005 79

Eligible for FASD diagnosis Canadian guidelines 2015 23

**Table 3:** The PAE ranks associated with specific diagnosis

	Alcohol Rank 2 (*n* = 29)	Alcohol Rank 3 (*n* = 84)	Alcohol Rank 4 (*n* = 32)	Total (*n* = 145)*
Fetal Alcohol Syndrome (FAS)	0 (0%)	1 (1.2%)	0 (0%)	1 (0.7%)
FAS/ Alc Exposed (AE) Unknown	1 (3.4%)	0 (0%)	0 (0%)	1 (0.7%)
Partial FAS	0 (0%)	6 (7.1%)	4 (12.5%)	10 (6.9%)
Static Encephalopathy (SE)/ AE	0 (0%)	49 (58.3%)	19 (59.4%)	68 (46.9%)
SE/AE Unknown	14 (48.3%)	0 (0%)	0 (0%)	14 (9.7%)
*Neurodevelopmental Disorder (ND)/AE*	0 (0%)	28 (33.3%)	9 (28.1)	37 (25.5%)
*ND/AE Unknown*	14 (48.3%)	0 (0%)	0 (0%)	14 (9.7%)

*1 study participant diagnosis was missing

### Implications of the PAE definition on the FASD diagnosis

**Fig. 3:**
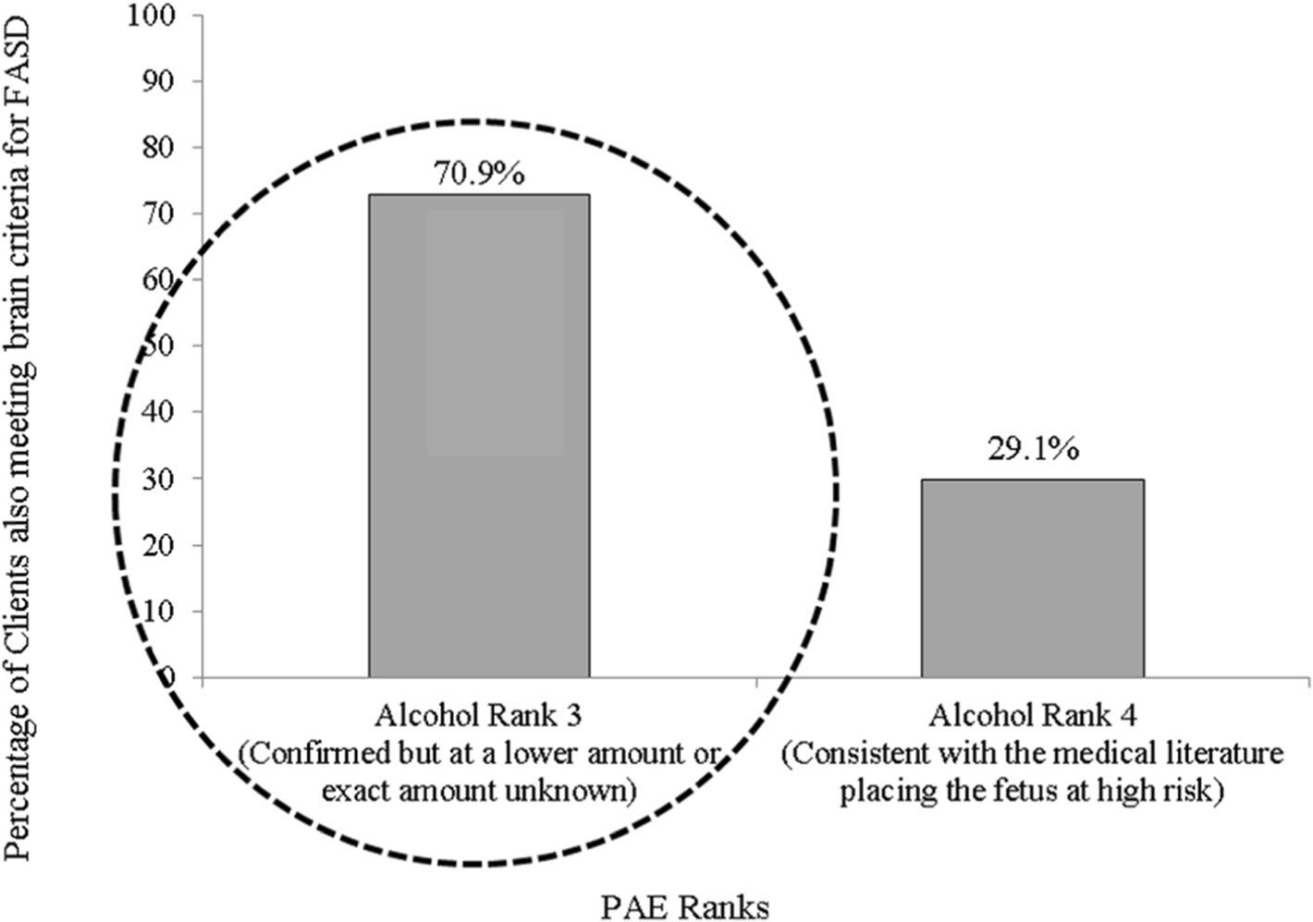
Percentage of clients meeting brain criteria for FASD (4-Digit Code Brain Rank 3 or 4) with reported prenatal alcohol exposure

In the present study 79 (54.1%) of the 146 clients got an FASD diagnosis based on their Brain rank of 3 or 4 plus their PAE rank of 3 or 4 (see Table 2). However, when the more stringent PAE criteria in 2015 Guidelines are applied, out of 79 FASD diagnoses made, only 23 (29.1%) would hold their diagnosis whereas 56 (70.9%) clients with previous FASD diagnosis, would now NOT be eligible for an FASD diagnosis (see Fig. 3). Furthermore, one client with full FAS and 6 clients with Partial FAS would also lose their diagnoses (see Table 3). The percentage not meeting the FASD diagnostic criteria under the new Guidelines using a more stringent PAE criteria are encircled in Fig. 3.

### Part-2 qualitative

In part-2 challenges faced by the FASD diagnostic team and community social workers in getting a credible PAE and the strategies implemented to collect a PAE were examined.

Following a qualitative research design, 30 min semi-structured interviews were conducted with 6 clinicians of the FASD diagnostic team at CYS in Regina plus their manager. Of the 30 social workers from Regina and rural southern Saskatchewan invited via email 17 participated voluntarily. All interviews were audio recorded and transcribed verbatim.

### Analysis

Transcribed interviews were analyzed qualitatively using QSR NVivo® 9 (© QSR International, 2011). The data was analysed employing Miles, Huberman and Saldana methods of qualitative data analysis [14]. All transcripts were reviewed line-by-line and coded into distinct categories in order to break the data down into the smallest meaningful units. Categories were constructed by grouping together base-level codes sharing similar ideas. This process of joining categories of data together continued until broad themes were identified. Transcripts was analyzed by one researcher and analysis resulted in 44 unique coding classifications, which were combined into 20 categories and finally clustered under three broad thematic areas described below.

## Results

### Value of assessment and diagnosis

Participants interviewed indicated that assessment and diagnosis was important to connect patients with appropriate supports and services. The professionals interviewed were unaware of any comprehensive services specifically dedicated to FASD in Saskatchewan. However, they have observed that a FASD diagnosis helped get additional school supports, teaching assistants and one-on-one staff to support the learning needs, as a participant mentioned.

“if a kid gets diagnosed with FASD, then there’s different funding so that the schools can maybe get some more-Educational Assistants to work with kids or different supports.”

Assessment and diagnosis was perceived to steer the focus towards appropriate management and finding supports for the child reducing frustration when there were behaviour challenges.

### Prenatal alcohol history

Many social workers were unaware of the PAE history details required in the 2015 guidelines. Once made aware of the 2015 requirements the professionals felt it would be challenging to obtain such detailed PAE history as is evident from this participants response *“Asking the birth mother if possible, where possible … ..You still may not get reliable information”.* The absence of the birth mother, the unwillingness of a birth mother to give a PAE history due to stigma and the fear that this information could jeopardize access or custody, was felt to influence the extent to which reliable PAE history can be collected. In the absence of history from the birth mother, information is often requested from reliable extended family members although the credibility of those sources is difficult to assess. Participants mentioned using third party sources such as police record of arrests, emergency hospital visits, visits to detox centers during pregnancy and birth records accessed especially for a biological mother with a known history of substance abuse.

A significant number of clients referred for FASD diagnosis are from disadvantaged families, Indigenous communities and those in the care or with the Ministry of Social Services. It was observed that very few referred clients come from white middle class families which was puzzling given the fact that alcohol use is common in all sectors of the local population.

### Effective strategies for obtaining PAE history

Participants feel that all healthcare professionals, parents, guardians etc. should be informed about the importance of asking about PAE in pregnancy and or recording the PAE history whenever the situation arises, in a way that the information could be used for future FASD assessments. When discussing PAE focus should be on gathering credible information required for diagnosis and assessment rather than emphasising the behaviour of the biological mother as a participant mentioned

*“when you work with families that are struggling with whatever issues are with their child, so when you are connecting them with the community supports, they are more willing to open up and discuss those things [PAE].”*

Online resources for FASD, effective behaviour management strategies and a list of available services can be valuable resources for professionals and caregivers of children with FASD in small towns and rural areas.

## Discussion

When the higher PAE threshold suggested in the 2015 Guidelines was re-applied to all the FASD assessments that were previously completed following the 2005 Guidelines from one FASD diagnostic clinic, a major drop was observed in the number of patients who could get an FASD diagnosis. This drop was observed even though those clients met Brain criteria for FASD and had PAE confirmation. The reason for this drop was simply the consequence of setting a minimum threshold for PAE proposed in the 2015 Canadian Guidelines Appendix.

This study demonstrates that diligent adherence to the minimal PAE threshold suggested in the 2015 Canadian Guidelines for FASD Diagnosis, can reduce the number diagnosed with FASD dramatically. We anticipate that this finding would be replicated in other Canadian FASD diagnostic clinics. This can lead to further under diagnosis precluding patients from much needed early interventions required to mitigate future neurodevelopmental problems [15].

The PAE history is crucial to initiate an FASD assessment [8, 11, 16]. Experienced professionals recognize that an accurate PAE history is exceedingly difficult to obtain. Even when the PAE history is available, the accuracy of the information cannot be verified due to factors such as mothers under reporting alcohol use [17], recall bias, second hand information, reliance on distant memory in adult assessments, social factors and stigma, changes in drink sizes with situation and location at which drinks were consumed and lack of questionnaires capturing the amount, pattern and time of alcohol consumption. Anecdotally clinicians report dads inflating mom’s alcohol use during child custody issues, or a mother reporting higher use to mitigate legal action against her child. Lack of training, knowledge and attitudes towards PAE may preclude healthcare professionals from actively recording PAE during medical history intake [18]. Survey of frontline professionals gathering PAE history, reported that it would be impractical if not impossible in most cases to obtain a detailed PAE history. Consistent with the literature they reported being guided by “common sense” or their own experience when gathering PAE histories [19]. Although PAE history is essential for FASD, standardized or validated screening tools or guidelines for obtaining PAE is lacking [20]. It is rather interesting to note that although it is common knowledge that PAE history is difficult to obtain, limited efforts have been employed to develop PAE assessment tools, standardised PAE history intake, mandate PAE history intake during prenatal care and birth histories or train clinicians and frontline staff to reliably record PAE history. A modified version of the Timeline Follow Back Procedure (TFBP), a gold standard tool for assessing alcohol exposure^6^ was successfully field tested to gather PAE in *pregnant* women [21]. Development of a reliable and validated tool to gather PAE history from past pregnancies could immensely facilitate more accurate and reliable PAE history intake ensuring diagnostic validity and allow across clinic comparisons.

Setting a PAE threshold assumes that the history *should* be obtainable, when the clinical reality is the opposite. Furthermore, a minimum PAE threshold implies that below threshold is “safe” or when high PAE is reported, the contribution of other prenatal and postnatal risk factors which significantly influences person’s neurodevelopmental disability maybe discounted [4, 22]. Setting a more stringent PAE criterion is incompatible with the realities of our diagnostic clinics and prevents accurate identification of PAE associated disorders.

As accurate and detailed PAE histories in clinical setting can rarely be gathered, the necessity of PAE threshold for an FASD diagnosis becomes questionable. Detailed PAE history is not required by the FASD 4-Digit Code which has 2 decades of data showing that there is substantial access that meets the needs of across the spectrum FASD population diagnosed under this system [23].

In Saskatchewan which has a population of about 1 million, 15,829 live births were reported in 2018 [24]. If the prevalence rate is accepted at 4.4% [25] then there should be an estimated 700 new FASD diagnosis per year. In our clinic which sees the southern half of the province 79 clients got an FASD diagnosis over the 6 year study period when the 2005 guidelines were implemented. Although our clinic provides diagnostic services to only the southern part of the province, it is very likely that a large number of cases are being missed and are not being connected to appropriate and timely care. If the 2015 guidelines are followed that would further reduce the number of clients who would receive FASD diagnosis.

Absence of PAE history creates a dilemma that for many of those clients the PAE levels were likely sufficient to have led to neurodevelopmental impairments. Many of the neurological and behavioural impairments observed in children with FASD resemble behaviours observed in other conditions such as ADHD, Learning disability, generalised anxiety disorder etc. Therefore it is likely in absence of adequate PAE history clients receive alternate diagnosis and are linked with therapies to manage those alternate conditions. However, FASD clients who received an alternate diagnosis of ADHD for example might respond differently to pharmacological and non-pharmacological ADHD treatments. The principal aim of FASD diagnosis and assessment is to gain a comprehensive idea of the neuro-behavioural impairments, distinguish cognitive or behavioural deficits among children with FASD from those due to other mental, neurological and developmental disorders and link clients with services appropriate to their diagnosis.

To achieve this, at our clinic the Canadian guidelines and the 4 digit codes are both utilized by the clinician during FASD diagnosis. Additionally a multi-disciplinary team carries a rigorous comprehensive assessment to reduce false positives and ensure behavioural impairments is due to the PAE and no other factors***.*** Although rigorous and comprehensive assessment is necessary for a complex condition like FASD, with the resources currently available at our clinic this type of approach is going to diagnose only a fraction of those with FASD.

### Recommendations

Emphasising a specific minimum level of PAE for an FASD diagnosis is problematic as has been demonstrated in this study. It is rather important to create awareness about FASD and emphasise the necessity of reliably documenting presence or absence of PAE in clinical settings among family physicians, pediatricians, neurologist, psychologists, prenatal care providers, social workers, addiction and mental health. Clinicians should be encouraged to obtain presence or absence of PAE information as part of standard prenatal care and birth histories. Emphasis should be given on ruling out FASD for all children entering care, entering juvenile corrections and for all children with a sibling diagnosed with FASD. Training should be offered to clinicians to gather PAE histories in a supportive and non-judgmental environment where emphasis is given on arriving at a conclusive diagnose and linking clients to appropriate services rather than dwelling on the behaviour of the biological mother. Frontline care providers should have training and support on how and when to obtain the PAE history and how and where to refer clients for an FASD assessment in a timely manner.

The interviews with social workers revealed that clinicians often use “third party sources, such as police record of arrests, emergency hospital visits, visits to detox centers during pregnancy and birth records” to obtain PAE histories which should be encouraged. Development of a reliable and validated tool to gather PAE history from past pregnancies could immensely facilitate more accurate and reliable PAE history intake ensuring diagnostic validity and allow across country comparisons.

### Study limitations

This study was conducted in one diagnostic clinic in southern Saskatchewan and might not be representative of all clinics across Canada. However, a similar trend is projected for other clinics using the same diagnostic systems. Only those social workers volunteering to participate were interviewed leading to possible sampling bias and the view might not be representative of all professionals referring for FASD assessments.

Canadian diagnostic guidelines permit an FASD diagnosis for those with Brain Rank of 3 or 4 compared to others countries in which Brain Rank 2 might qualify for FASD diagnosis. Therefore an even a larger number would lose their diagnosis.

Over the years, an increased number of mothers are participating in assessment and increased awareness about the required PAE details mentioned in the new guidelines may have improved PAE history intake, leading to increased number of PAE Rank 4 observed currently, compared to that reported in the present study.

## Conclusion

Unless it is demonstrated that an accurate and detailed PAE history is actually *possible* and it is clinically beneficial for patients, the new Canadian Guidelines should add an addendum of caution clarifying the most clinically practical definition of PAE so as not to deny patients an assessment and appropriate diagnosis needed to access services and supports. This will allow capture of the full epidemiological and clinical scope of the problem.

## Data Availability

The datasets during and/or analysed during the current study are available from the corresponding author on reasonable request.

## Abbreviations

ADHD: Attention-Deficit Hyperactivity Disorder
AE: Alc Exposed
CNS: Central Nervous System
CYS: Child and Youth Services
FAS: Fetal Alcohol Syndrome
FASD: Fetal Alcohol Spectrum Disorder
ND: Neurodevelopmental Disorder
PAE: Prenatal Alcohol Exposure
SE: Static Encephalopathy

## References

[1] Public Health Agency of Canada. Fetal Alcohol Spectrum Disorder (FASD). A framework for action. Ottawa: Public Health Agency of Canada. Available: https://www.canada.ca/content/dam/phac-aspc/migration/phac-aspc/publicat/fasd-fw-etcaf-ca/pdf/fasd-fw_e.pdf. Accessed 15 Oct 2017.

[2] Lange S, Probst C, Gmel G, Rehm J, Burd L, Popova S (2017). Global prevalence of fetal alcohol Spectrum disorder among children and youth: a systematic review and meta-analysis. JAMA Pediatr.

[3] Systematic review on the prevalence of fetal alcohol spectrum disorders. Institute of Health Economics. Available: http://fasd.alberta.ca/documents/Systematic_Prevalence_Report_FASD.pdf. Accessed Jan 2018.

[4] Hoyme H. Eugene, Kalberg Wendy O., Elliott Amy J., Blankenship Jason, Buckley David, Marais Anna-Susan, Manning Melanie A., Robinson Luther K., Adam Margaret P., Abdul-Rahman Omar, Jewett Tamison, Coles Claire D., Chambers Christina, Jones Kenneth L., Adnams Colleen M., Shah Prachi E., Riley Edward P., Charness Michael E., Warren Kenneth R., May Philip A. (2016). Updated Clinical Guidelines for Diagnosing Fetal Alcohol Spectrum Disorders. Pediatrics.

[5] National Screening Tool Kit for Children and Youth Identified and Potentially Affected by FASD. Canadian Association of Paediatric Health Centres. [Available: https://ken.caphc.org/xwiki/bin/view/FASDScreeningToolkit/National+Screening+Tool+Kit+for+Children+and+Youth+Identified+and+Potentially+Affected+by+FASD (accessed Jan 2018)].

[6] Popova S, Lange S, Probst C, Gmel G, Rehm J (2017). Estimation of national, regional, and global prevalence of alcohol use during pregnancy and fetal alcohol syndrome: a systematic review and meta-analysis. Lancet Glob Health.

[7] Bakhireva LN, Garrison L, Shrestha S, Sharkis J, Miranda R, Rogers K (2018). Challenges of diagnosing fetal alcohol spectrum disorders in foster and adopted children. Alcohol.

[8] Diagnostic Guide for Fetal Alcohol Spectrum Disorders: the 4-Digit Diagnostic Code. Available: https://depts.washington.edu/fasdpn/pdfs/guide04.pdf. Accessed Mar 2018.

[9] Astley SJ (2013). Validation of the fetal alcohol spectrum disorder (FASD) 4-Digit Diagnostic Code. J Popul Ther Clin Pharmacol.

[10] Astley SJ, Clarren SK (2000). Diagnosing the full spectrum of fetal alcohol-exposed individuals: introducing the 4-digit diagnostic code. Alcohol Alcohol.

[11] Cook JL, Green CR, Lilley CM, Anderson SM, Baldwin ME, Chudley AE, Conry JL, LeBlanc N, Loock CA, Lutke J (2016). Fetal alcohol spectrum disorder: a guideline for diagnosis across the lifespan. CMAJ.

[12] Benz J, Rasmussen C, Andrew G (2009). Diagnosing fetal alcohol spectrum disorder: history, challenges and future directions. Paediatr Child Health.

[13] Hemingway SJA, Bledsoe JM, Davies JK, Brooks A, Jirikowic T, Olson EM, Thorne JC. Twin study confirms virtually identical prenatal alcohol exposures can lead to markedly different fetal alcohol spectrum disorder outcomesfetal genetics influences fetal vulnerability. Adv Pediatr Res. 2019;5(23):1-19. 10.24105/apr.2019.5.23 PMC7757639 33364429

[14] Mathew B, Miles MH, Saldana J. Qualitative data analysis: a methods sourcebook, fourth edn. Thousand Oaks: SAGE Publications Inc.; 2019.

[15] Streissguth AP, Bookstein FL, Barr HM, Sampson PD, O'Malley K, Young JK (2004). Risk factors for adverse life outcomes in fetal alcohol syndrome and fetal alcohol effects. J Dev Behav Pediatr.

[16] Chudley AE, Conry J, Cook JL, Loock C, Rosales T, LeBlanc N (2005). Fetal alcohol spectrum disorder: Canadian guidelines for diagnosis. CMAJ.

[17] Ernhart CB, Morrow-Tlucak M, Sokol RJ, Martier S (1988). Underreporting of alcohol use in pregnancy. Alcohol Clin Exp Res.

[18] Payne JM, France KE, Henley N, D'Antoine HA, Bartu AE, O'Leary CM, Elliott EJ, Bower C, Geelhoed E (2011). RE-AIM evaluation of the alcohol and pregnancy project: educational resources to inform health professionals about prenatal alcohol exposure and fetal alcohol spectrum disorder. Eval Health Prof.

[19] Brown NN, Burd L, Grant T, Edwards W, Adler R, Streissguth A (2015). Prenatal alcohol exposure: an assessment strategy for the legal context. Int J Law Psychiatry.

[20] Goh YI, Chudley AE, Clarren SK, Koren G, Orrbine E, Rosales T, Rosenbaum C (2008). Development of Canadian screening tools for fetal alcohol spectrum disorder. Can J Clin Pharmacol.

[21] Dukes K, Tripp T, Petersen J, Robinson F, Odendaal H, Elliott A, Willinger M, Hereld D, Raffo C, Kinney HC (2017). A modified Timeline Followback assessment to capture alcohol exposure in pregnant women: Application in the Safe Passage Study. Alcohol.

[22] Astley SJ, Bledsoe JM, Davies JK, Thorne JC (2017). Comparison of the FASD 4-digit code. FASD diagnostic guidelines. Adv Pediatr Res.

[23] Astley SJ (2014). The value of a FASD diagnosis (2013). J Popul Ther Clin Pharmacol.

[24] Number of births in Saskatchewan, Canada in fiscal years 2001 to 2018. [Online]. https://www.statista.com/statistics/578587/number-of-births-in-saskatchewan-canada/. Accessed July 2019.

[25] Flannigan K, Unsworth, K, Harding, K. The Prevalence of Fetal Alcohol Spectrum Disorder. 2018; CanFASD. [Online]. https://canfasd.ca/wp-content/uploads/sites/35/2018/08/Prevalence-1-Issue-Paper-FINAL.pdf. Accessed July 2019.

